# 1554. Facilitators and Barriers to Engaging in PrEP and Gender Affirming Therapy for Black and Latina Transgender Women in South Florida

**DOI:** 10.1093/ofid/ofad500.1389

**Published:** 2023-11-27

**Authors:** Rebe Silvey, Yesenia Rosas, Kecia L Ellick, Ariana Johnson, Lacey Craker, Susanne Doblecki Lewis, Suzanne Randolph Cunningham, Aliya F Rehman, Mariano Kanamori, Laura Beauchamps

**Affiliations:** University of Miami, Miller School of Medicine, coral gables, Florida; University of Miami, Miller School of Medicine, coral gables, Florida; KLE Consulting, Atlanta, Georgia; University of Miami, miami, Florida; University of Miami, miami, Florida; University of Miami, Miller School of Medicine, coral gables, Florida; The MayaTech Corporation, College Park, Maryland; University of Miami, miami, Florida; University of Miami Miller School of Medicine, Miami, Florida; University of Miami Miller School of Medicine, Miami, Florida

## Abstract

**Background:**

Black and Latina transgender women (BLTW) have the highest HIV rates among transgender women (Black: 62%, Latinx: 35%). PrEP-GAT is an EHE grant which evaluated barriers and facilitators to engagement in PrEP and gender affirming therapy (GAT) services and the use of friendship networks to promote these interventions.

**Methods:**

Participants were recruited through the University of Miami’s GenWell Service, which provides bundled PrEP and GAT. This study has two components: qualitative and social networks. Qualitative component: 20 in-depth interviews focused on barriers and facilitators to PrEP and GAT services using Consolidated Framework for Implementation Research (CFIR) constructs. Data was analyzed using thematic analysis. Social network component: 27 social network-based interviews identified friendship dynamics that could promote conversations and encouragement to engage with PrEP and GAT. Analysis included multilevel logistic regression using R and network visualizations using UCINET.

**Results:**

Qualitative Component. Barriers to accessing PrEP and GAT services included cost, need for Spanish-language materials, bias, stigma, and discrimination. Facilitators included access to telehealth, mobile services, pharmacists, and co-located PrEP-GAT services. BLTW were not comfortable disclosing their HIV status due to HIV stigma. “HIV clinics” discouraged individuals from seeking services. Social network component: most participants were using GAT (75%), and more than half reported lifetime PrEP use (56%). Bivariate analysis: having a Latinx friend, a friend who shared GAT status, and higher emotional closeness were associated with future conversations about PrEP and GAT (*p*< 0.05). Multilevel model: emotional closeness was associated with future conversations about PrEP and GAT (*p*=0.02).

**Conclusion:**

Mental health, legal, employment and housing services are needed to meet BLTW access to PrEP. Social network approaches can identify key individuals in BLTW friendship networks to promote and disseminate information about PrEP and GAT.

Future Directions: Future research will determine whether bundled PrEP-GAT, social network support, and telehealth is effective to increase BLTW’s engagement in these services.

**Disclosures:**

**Susanne Doblecki Lewis, MD, MSPH, FIDSA**, Gilead Sciences: Grant/Research Support|Janssen: Grant/Research Support|Merck: Grant/Research Support

